# Carotid baroreflex responsiveness in normotensive African Americans is attenuated at rest and during dynamic leg exercise

**DOI:** 10.3389/fphys.2013.00029

**Published:** 2013-03-08

**Authors:** Seth W. Holwerda, Mitchel R. Samels, David M. Keller

**Affiliations:** ^1^Department of Medical Pharmacology and Physiology, University of MissouriColumbia, SC, USA; ^2^Department of Kinesiology, University of Texas ArlingtonArlington, TX, USA

**Keywords:** blood pressure, heart rate, racial differences, hypertension, exercise

## Abstract

Evidence suggests differences between African Americans (AAs) and Caucasian Americans (CAs) in cardiovascular responsiveness to physiological stressors. This study tested the hypothesis that carotid baroreflex (CBR) control of heart rate (HR) and blood pressure is reduced in AAs compared to CAs during exercise. Mean arterial pressure (MAP) and HR were continuously recorded at rest and during leg cycling in 23 non-hypertensive male subjects (12 AA; 11 CA; age 19–26 years). CBR control of HR and MAP was assessed with 5-s pulses of neck pressure (NP, simulated hypotension) and neck suction (NS, simulated hypertension) ranging from +45 to −80 Torr. Across all NS stimuli (−20, −40, −60, −80 Torr) at rest, the AA group demonstrated attenuated CBR-mediated reductions in HR (AA, −8.9 ± 1.9 vs. CA, −14.1 ± 2.3 bpm; *P* < 0.001) and MAP (AA, −6.4 ± 1 vs. CA, −7.8 ± 0.8 mmHg; *P* < 0.05). Despite similar gain and magnitude of resetting observed in the modeled stimulus response curves, an attenuation among AAs persisted in HR (AA, −8.2 ± 1.6 vs. CA, −11.8 ± 3 bpm; *P* < 0.05) and MAP (AA, −6.8 ± 0.9 vs. CA, −8.2 ± 1.1 mmHg; *P* < 0.05) responses to NS during exercise. No differences in CBR-mediated HR and MAP responses to NP were detected between groups at rest or during exercise. These data suggest impairment in the ability to defend against a hypertensive challenge among AAs during steady-state exercise compared to their CA counterparts.

## Introduction

Hypertension affects African Americans (AAs) at a rate that is among the highest in the world (Lloyd-Jones et al., 2010). AAs are at 1.8X greater risk of fatal stroke related to hypertension and are over 4X more likely to develop end-stage renal disease associated with hypertension (NHLBI, 2004). Accumulating evidence indicates augmented blood pressure responses to emotional and physical stimuli in AAs compared to Caucasian American (CAs), (Light et al., 1987; Anderson et al., 1988; Treiber et al., 1990; Calhoun et al., 1993; Terrell and Manuck, 1996; Calhoun and Mutinga, 1997; Barnes et al., 2000; Kelsey et al., 2000; Bond et al., 2001; Arthur et al., 2004). AAs also exhibit exaggerated blood pressure responses to exercise relative to CAs (Alpert et al., 1981; Thomas et al., 1987; Ekelund et al., 1990; McAdoo et al., 1990; Walker et al., 1992; Duey et al., 1997). While many studies support racial differences for blood pressure responses to the aforementioned stimuli, mechanisms associated with dynamic reflex control of blood pressure (e.g., arterial baroreflex function) have not been thoroughly investigated.

Regulation of beat-to-beat changes in arterial blood pressure is largely determined by arterial baroreflex-mediated changes in autonomic neural activity to the heart and vasculature. Some differences between AAs and CAs in neural control of arterial blood pressure during rest have been reported (Ray and Monahan, 2002; Franke et al., 2004; Hinds and Stachenfeld, 2010; Holwerda et al., 2011). In addition to blunted declines in cardiac output and total peripheral conductance in AA compared to CA in response to baroreceptor unloading (i.e., lower-body negative pressure) at rest (Franke et al., 2004), augmented transduction of sympathetic nerve activity to vascular resistance in AAs compared to CAs has also been reported under similar conditions (Ray and Monahan, 2002). We recently reported impaired carotid baroreflex (CBR) responsiveness among AAs compared to CAs (Holwerda et al., 2011). Our primary observation was a distinct impairment in CBR-mediated reductions in heart rate (HR) in response to simulated hypertension among AAs at rest. However, it is not clear how the potential racial differences in arterial baroreflex function may manifest during exercise, a condition in which the arterial baroreflex plays an important role in the regulation of cardiovascular adjustments.

An intact arterial baroreflex is essential for appropriate blood pressure responses to exercise (Melcher and Donald, 1981; Walgenbach and Donald, 1983; Walgenbach and Shepherd, 1984; Scherrer et al., 1990). Resetting of the reflex during exercise allows for continued control of arterial blood pressure across a wide spectrum of exercise intensity (Potts et al., 1993; Papelier et al., 1994; Fadel and Raven, 2012). While greater blood pressure responses have been reported in AA compared to CA during dynamic (Alpert et al., 1981; Thomas et al., 1987; Ekelund et al., 1990; Walker et al., 1992) and static exercise (McAdoo et al., 1990; Duey et al., 1997), inadequate baroreflex buffering of exercise-induced increases in blood pressure, such as those mediated by the exercise pressor reflex, would theoretically lead to inappropriate cardiovascular responses [e.g., inadequate blood flow distribution due to inadequate baroreflex buffering of the sympathetic neural outflow (Joyner, 2006)].

This study tested the hypothesis that CBR control of HR and blood pressure is reduced in AAs compared to CAs during exercise. To test this hypothesis, we examined CBR-mediated changes in HR and Mean arterial pressure (MAP) to wide range of simulated hypertensive and hypotensive stimuli at rest and during dynamic leg cycle exercise in normotensive AAs, and age, BMI and fitness-matched normotensive AAs.

## Methods

### Subjects

Twenty three adult, non-hypertensive male subjects (12 AAs; 11 CAs; age 19–26 years) were recruited from the University of Texas at Arlington. All subjects were free of known cardiovascular and respiratory diseases, non-smokers, and recreationally active (low to moderate intensity activity). Following the recruitment of AA subjects, CA subjects [i.e., similar fitness (VO_2MAX_ within 7 ml/kg/min), age (within 3 years) and body mass index (within 15%)] were recruited. 8 of the 12 AAs and 4 of the 11 CAs have previously volunteered as research subjects in our laboratory (Holwerda et al., 2011). Completely new experiments were performed on the previously studied volunteers. Each subject signed an informed consent that was approved by the Institutional Review Boards at the University of Texas at Arlington. Prior to participation, all subjects were familiarized with the testing protocols. Subjects were advised to not consume alcohol within the 24 h prior and not to consume caffeine within 12 h prior to the scheduled experiment. Subjects were also advised not to vigorously exercise for 48 h prior to the scheduled experiment. Subjects were not using prescription or over-the-counter medications, and known family history of hypertension was recorded for all subjects.

### Prior to experimentation

Each subject completed a medical health history questionnaire and a resting blood pressure screening. Subjects underwent a maximal exercise test on a recumbent leg cycle ergometer while obtaining continuous measurements of respiratory gases (TrueOne 2400, Parvo Medics) to establish maximal oxygen uptake for the determination of cardiorespiratory fitness and steady-state exercise workloads. Due to variability in the anatomical location of the carotid sinus, each subject's carotid sinuses were confirmed as appropriate for the neck chamber by Doppler ultrasound. Resting trials of NP and NS were performed for familiarization with the experimental measurements and protocol.

### Experimental measurements

Subjects were instrumented with a 3-lead electrocardiogram (ECG) and an automated sphygmomanometer (Tango^+^, Suntech) for continuous HR and steady-state arterial blood pressure measurements, respectively. Beat-to-beat arterial blood pressure was measured using a servo-controlled finger-cuff photoplethysmograph (Finometer® Pro, Finapres Medical Systems, Amsterdam, Netherlands) from a finger on the right hand while it rested at the level of the right atrium. Beat-to-beat arterial blood pressure recordings were adjusted to absolute steady-state arterial blood pressure measurements as determined by an automated sphygmomanometer, which has been validated for use during dynamic exercise (Cameron et al., 2004). Continuous measurements of respiratory gases (TrueOne 2400, Parvo Medics) were taken during the exercise phase of the experiment. CBR control of HR and MAP was assessed through the use of 5-s periods of neck pressure (NP, simulated hypotension) and neck suction (NS, simulated hypertension) delivered to the region of the carotid sinuses encased by a properly sized malleable neck chamber. Pressure and suction pulses were generated by a variable pressure source and delivered to the neck chamber through two-way solenoid valves and controlled using custom software (NS3). Data were sampled at 200 Hz and stored for off-line analysis (AcqKnowledge, BioPac Systems Inc., Goleta, CA).

### Experimental procedures

#### Maximal exercise test

Subjects were seated in a semirecumbent position on an examination table equipped with an electrically braked cycle ergometer (Corival Supine, Lode, Groningen, The Netherlands), and instrumented with a 3-lead ECG and an automated arterial blood pressure cuff. After a 3-min warm-up at 50 Watts and preferred pedal frequency between 50 and 70 rpm, exercise workload increased 25 Watts each minute until the subject could no longer maintain pedal frequency. All subjects received verbal encouragement, and reported a rating of perceived exertion at test termination.

#### Experimental day

Subjects returned for experimental measures after at least 48 h following the maximal exercise test. Subjects were instrumented with a 3-lead ECG, automated arterial blood pressure cuff, and a finger-cuff photoplethysmograph. After 20 min of supine rest, subjects were seated in a semi-recumbent position (~60°upright) and were fitted with a properly sized malleable neck chamber.

CBR responsiveness was assessed by applying multiple trials of random-ordered single 5-s pulses of NP and NS ranging from +45 to −80 Torr (i.e., +45, +30, +15, −20, −40, −60, −80). Each pressure stimulus was delivered to the carotid sinus during a 15 s breath hold at normal end-expiration to minimize respiratory-related modulation of HR and MAP. The generated pressures within the neck collar were manually controlled and a pressure transducer (model DP45, Validyne Engineering, Northridge, CA, USA) was connected to a port on the collar to accurately quantify the stimulus applied. At least four trials of each magnitude of NS and NP were administered with a minimum of 45 s of recovery between trials to allow variables to return to pre-stimulus values (Ogoh et al., 2003b).

The electrically braked cycle ergometer was positioned for the exercise bout following the resting NP and NS trials. After a 5-min warm-up at 50 Watts and a preferred pedal frequency between 50 and 70 rpm, workload was adjusted to elicit steady-state oxygen consumption levels of ~50% of maximal oxygen uptake. To minimize the potential of cardiovascular drift, only 3–4 trials at each chamber pressure were performed during exercise (Norton et al., 1999). The end-expiratory breath hold was not performed during exercise to diminish the potential for chemoreflex activation.

### Data analysis

#### Absolute steady-state blood pressure measures

In order to account for the change in the systolic/diastolic period ratio as HR increases, absolute steady-state MAP values at rest and during exercise were calculated as a function of HR in place of the standard MAP equation [MAP = DBP + 1/3(SBP−DBP)] (Moran et al., 1995). First, the fraction of systole (St) of the cardiac cycle was related to HR using the following equation: St = 0.01exp (4.14–40.74/HR). Diastolic blood pressure (DBP) and pulse pressure (PP) were then adjusted for St in the following equation: MAP = DBP + St (PP).

#### Maximum MAP and HR responses

The maximum MAP response was determined by assessing the three cardiac cycle interval with the largest change in MAP relative to pre-stimulus (three cardiac cycle average) for each trial of NP and NS. This analysis has been described previously (Keller et al., 2006). For HR, the single maximum cardiac cycle response was compared to pre-stimulus (three cardiac cycle average) for each trial of NP and NS.

#### Carotid baroreflex function curves

Carotid-cardiac and carotid-vasomotor stimulus-response curves were determined by plotting the maximal changes in HR and MAP, respectively, elicited by NP and NS against the estimated carotid sinus pressure (ECSP), which was calculated as mean blood pressure minus neck chamber pressure. CBR stimulus-response data were fit for each subject to the logistic function model described by Kent et al. (1972): Dependent variable = A_1_ {1 + exp[A_2_(ECSP-A_3_)]}^−1^ + A_4_ where the dependent variable is HR or mean blood pressure, A_1_ is the range of response of the dependent variable (maximum–minimum), A_2_ is the gain coefficient, A_3_ is the centering point or carotid sinus pressure required to elicit equal pressor and depressor responses, and A_4_ is the minimum response.

The CBR operating point gain and maximal gain were calculated using the equations: G_op_ = A_1_A_2_exp [A_2_(ECSP_op_ – A_3_)]/{1 + exp[A_2_(ECSP_op_ – A_3_)]}^2^ and G_max_ = −A_1_A_2_/4 where G_op_ is the gain of the CBR function curve at the operating point, G_max_ is the maximal gain of the CBR function curve, and ECSP_op_ is the ECSP at the operating point (i.e., prestimulus MAP). The G_op_ was calculated as the gain at the operating point and used to provide a measure of responsiveness at the operating point of the CBR function curve, whereas the G_max_ was calculated as the gain at the centering point and used as an index of overall CBR responsiveness. The threshold (THR) and saturation (SAT), described as the minimum and maximum ECSP, respectively, that elicits a reflex change in HR or MAP, were calculated using the following equation: THR = −2.944/A_2_ + A_3_ and SAT = 2.944/A_2_ + A_3_. The parameters for all subjects within an experimental condition were averaged to provide group mean responses.

The movement of the operating point away from the centering point was described by the following equation: OP-CP. The magnitude of baroreflex resetting during exercise (i.e., the upward and rightward movement of the carotid-cardiac and carotid-vasomotor curves) was determined by the sum of the changes in *A*_3_, *A*_4_, THR, and SAT from rest to exercise for each subject.

### Statistical analysis

Comparisons for changes in HR and BP from rest to exercise and descriptive characteristics (e.g., height, weight, BMI, VO_2MAX_), were made between racial groups using unpaired *t*-tests. The statistical comparison of the baroreflex and cardiovascular response variables between racial populations (factor 1) and the various magnitudes of NP and NS (factor 2) at rest and exercise were made using a Two-Way ANOVA. For comparison of carotid-cardiac and carotid-vasomotor response curve parameters between racial groups and conditions (i.e., rest vs. exercise), Two-Way ANOVA was used. Two-Way analysis of covariance (ANCOVA) was also used to determine if differences existed between racial groups after controlling for known family history of hypertension. Each subject's corresponding covariate data were determined as either negative (no parental hypertension) or positive (one or more cases of parental hypertension). Family history of hypertension was determined for all subjects. When required, multiple comparison procedures were performed using the Holm–Sidak method. Statistical significance was set at *P* < 0.05.

## Results

Subject characteristics are described in Table 1. There were no significant group differences in age, height, weight, VO_2_max, and BMI between racial groups. There were also no group differences in changes in HR, SBP, DBP, and MAP from rest to exercise.

**Table 1:** **Subject characteristics**.

	**AA (*n* = 12)**	**CA (*n* = 11)**	***P*-value**
Age (yr)	22 ± 2.3	22 ± 1.1	0.587
Height (m)	1.77 ± 0.08	1.81 ± 0.07	0.360
Weight (kg)	78.5 ± 13.4	78 ± 7.7	0.581
VO_2_max (ml/kg/min)	40.1 ± 7	42.9 ± 7.7	0.350
BMI (kg/m^2^)	25 ± 4	23.9 ± 1.8	0.428
ΔHR (bpm)	62 ± 3.9	64 ± 3.2	0.618
ΔSBP (mmHg)	49 ± 5.6	44 ± 3.8	0.404
ΔDBP (mmHg)	2.1 ± 3.4	−4.6 ± 3.6	0.144
ΔMAP^1^ (mmHg)	18 ± 2.9	12 ± 2.8	0.100
ΔMAP^2^ (mmHg)	29 ± 3.3	24 ± 3.3	0.232
Family history HTN	6(+), 6(−)	4(+), 7(−)	–

*Values expressed as mean ± SD*.

*AA, African American; CA, Caucasian American; VO_2_MAX, maximum oxygen consumption; BMI, body mass index; ΔHR, change in heart rate from rest to exercise, ΔSBP, change in systolic blood pressure from rest to exercise; ΔDBP, change in diastolic blood pressure from rest to exercise; ΔMAP^1^, change in mean arterial pressure from rest to exercise {DBP + [(SBP – DBP)/3]}; ΔMAP^2^, change in mean arterial pressure from rest to exercise corrected for systolic/diastolic period ratio (see “Methods”); HTN, hypertension*.

### Carotid baroreflex responsiveness at rest

**Figure 1:**
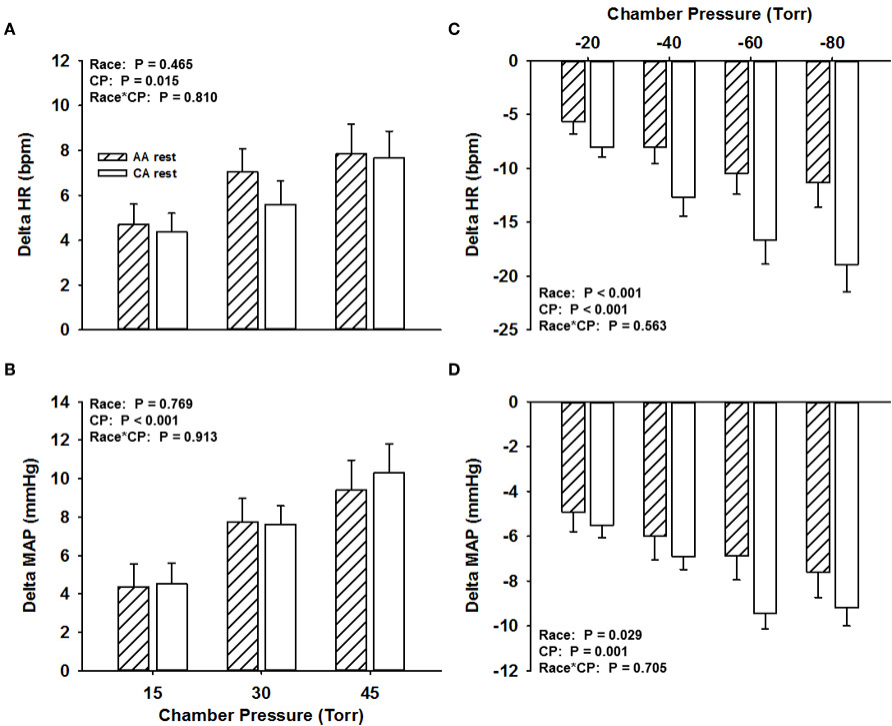
**Change in HR (A) and MAP (B) in response to NP and change in HR (C) and MAP (D) in response to NS in African Americans (AA, hatched bars) and Caucasian Americans (CA, open bars) at rest.** CP, chamber pressure.

Across all NP stimuli (15, 30, and 45 Torr), no significant differences (*P* > 0.05) were found in the magnitude of the HR responses (average value for all HR responses to NP stimuli at all pressures, AA, 6.5 ± 1.2 bpm; CA, 5.9 ± 1.1 bpm; Figure 1A) and MAP responses (AA, 7.2 ± 1.4 mmHg; CA, 7.5 ± 1.4 mmHg; Figure 1B). Similar findings were observed for HR and MAP responses to NP when controlling for family history of hypertension (Two-Way ANCOVA, both *P* > 0.05).

The magnitude of the HR response across all NS stimuli was attenuated in the AA group (−8.9 ± 1.9 bpm) compared to the CA group (−14.1 ± 2.3 bpm; *P* < 0.001; Figure 1C). The magnitude of the MAP response across all NS stimuli was also attenuated in the AA group (−6.4 ± 1 mmHg) compared to the CA group (−7.8 ± 0.8 mmHg; *P* < 0.05; Figure 1D). Similar findings were observed for HR and MAP responses to NS when controlling for family history of hypertension (Two-Way ANCOVA, both *P* < 0.05), despite a main effect of family history.

### Carotid baroreflex responsiveness during exercise

**Figure 2:**
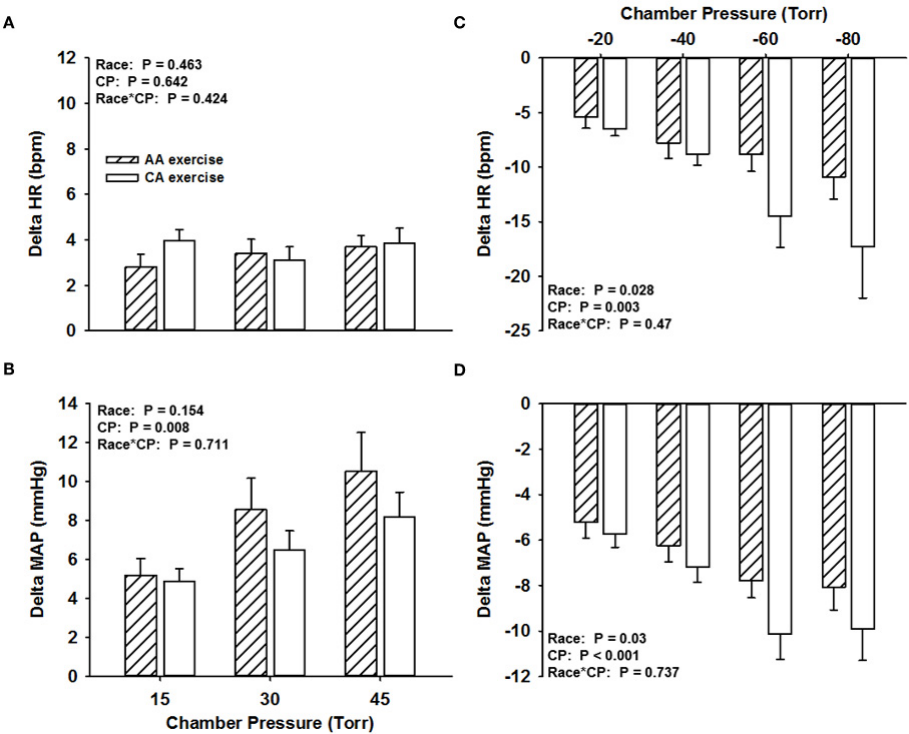
**Change in HR (A) and MAP (B) in response to NP and change in HR (C) and MAP (D) in response to NS in African Americans (AA, hatched bars) and Caucasian Americans (CA, open bars) during exercise.** CP, chamber pressure.

Across all NP stimuli, no significant differences (*P* > 0.05) were found in the magnitude of the HR responses (AA, 3.3 ± 0.6 bpm; CA, 3.6 ± 0.6 bpm; Figure 2A) and MAP responses (AA, 8.1 ± 1.6 mmHg; CA, 6.5 ± 1 mmHg; Figure 2B). Similar findings were observed for HR and MAP responses to NP when controlling for family history of hypertension (Two-Way ANCOVA, both *P* > 0.05).

The magnitude of the HR response across all NS stimuli during exercise was attenuated in the AA group (−8.2 ± 1.6 bpm) compared to the CA group (−11.8 ± 3 bpm; *P* < 0.05; Figure 2C). The magnitude of the MAP response across all NS stimuli during exercise was also attenuated in the AA group (−6.8 ± 0.9 mmHg) compared to the CA group (−8.2 ± 1.1 mmHg; *P* < 0.05; Figure 2D). Similar findings were observed for HR and MAP responses to NS when controlling for family history of hypertension (Two-Way ANCOVA, both *P* < 0.05), despite a main effect for family history for only MAP responses.

### Carotid-cardiac and carotid-vasomotor stimulus-response curves

**Figure 3:**
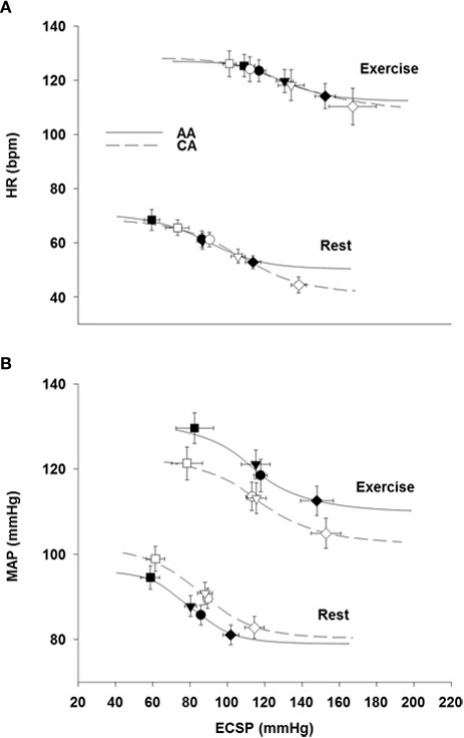
**Modeled carotid-cardiac (A) and carotid-vasomotor (B) baroreflex function curves at rest and during exercise in African Americans (AA, solid lines) and Caucasian Americans (CA, dashed lines).** ECSP, estimated carotid sinus pressure. Filled symbols represent AA and open symbols represent CA. Circles represent operating points, inverted triangles represent centering points, squares represent carotid sinus pressure threshold, rotated squares represent carotid sinus pressure saturation.

Table 2 describes the logistic model parameters and derived variables that describe CBR control of HR (carotid-cardiac) and MAP (carotid-vasomotor) at rest and during exercise in both groups. The carotid-cardiac *A*_3_, SAT, and magnitude of OP-CP was significantly less (*P* < 0.05) in the AA group compared to the CA group. Carotid-cardiac *A*_1_ (responding range) in the AA group tended to be diminished (Two-Way ANOVA, *P* = 0.059), reaching a statistically significant difference when controlling for family history of hypertension (Two-Way ANCOVA, *P* = 0.028). No differences (*P* > 0.05) were detected between groups in maximal or operating point gain for both carotid-cardiac and carotid-vasomotor. The significant increase in *A*_3_, *A*_4_, THR, and SAT from rest to exercise in both groups (all *P* < 0.05) describes the upward and rightward resetting of the carotid-cardiac (Figure 3A) and carotid-vasomotor (Figure 3B) stimulus response curves. The magnitude of resetting (i.e., sum of the changes in *A*_3_, *A*_4_, THR, and SAT from rest to exercise) was not different between groups (*P* > 0.05). Findings for all stimulus response curve variables other than carotid-cardiac *A*_1_ were similar when controlling for family history of hypertension (Two-Way ANCOVA).

**Table 2:** **Logistic model parameters and derived parameters**.

	**Rest**		**Exercise**	
	**AA**	**CA**	**AA**	**CA**
**CAROTID-CARDIAC CURVE**				
*A*_1_, bpm	20.5 ± 3.4	27.7 ± 3.6	14.8 ± 2.6	20.7 ± 4
*A*_2_, au	0.11 ± 0.04	0.07 ± 0.01	0.12 ± 0.02	0.08 ± 0.02
*A*_3_, mmHg^*^ ^†^	86.8 ± 3	106.2 ± 4	130.8 ± 4.6	134.3 ± 6.9
*A*_4_, bpm^†^	50.5 ± 2.3	40.2 ± 3.1	112.3 ± 4.7	107.8 ± 7.1
Threshold, mmHg^†^	59.7 ± 2.9	72.9 ± 6.5	109.2 ± 5.7	101.2 ± 3.6
Saturation, mmHg^*^ ^†^	113.8 ± 4	139.4 ± 4.1	152.5 ± 5.4	167.3 ± 12.6
G_max_, bpm/mmHg	−0.39 ± 0.05	−0.47 ± 0.07	−0.38 ± 0.06	−0.34 ± 0.05
G_op_, bpm/mmHg	−0.35 ± 0.05	−0.3 ± 0.04	−0.26 ± 0.05	−0.24 ± 0.04
OP-CP, mmHg^*^ ^†^	−0.49 ± 3.4	−15.9 ± 3.8	−13.8 ± 2.5	−21.8 ± 5.8
**CAROTID-VASOMOTOR CURVE**				
*A*_1_, mmHg	17.7 ± 2	20.8 ± 2	22.4 ± 3	21.7 ± 1.8
*A*_2_, au	0.12 ± 0.02	0.08 ± 0.01	0.08 ± 0.01	0.07 ± 0.02
*A*_3_, mmHg^†^	80.4 ± 3.3	89.7 ± 3.9	115.3 ± 7.8	116.1 ± 6
*A*_4_, mmHg^†^	79 ± 2.4	79.3 ± 2.5	109.9 ± 3.5	101.3 ± 3.8
Threshold, mmHg^†^	58.9 ± 5.1	62.4 ± 5.3	82.6 ± 10	78 ± 9.3
Saturation, mmHg^†^	102 ± 4.3	116.9 ± 5	148 ± 8.8	154.1 ± 8.8
G_max_, mmHg/mmHg	−0.49 ± 0.07	−0.41 ± 0.04	−0.42 ± 0.09	−0.36 ± 0.08
G_op_, mmHg/mmHg	−0.34 ± 0.06	−0.37 ± 0.04	−0.35 ± 0.07	−0.33 ± 0.08
OP-CP, mmHg	5.5 ± 2.1	−0.48 ± 2.7	3.2 ± 5.9	−3.2 ± 4.9

*Values expressed as mean ± SE*.

*AA, African American; CA, Caucasian American; A_1_, response range; A_2_, gain coefficient; A_3_, centering point; A_4_, minimum response; G_max_, maximal gain; G_op_, operating point gain; OP-CP, operating point-to-centering point; au, arbitrary units*.

* Main effect for race (P < 0.05);

† *Main effect of condition (rest vs. exercise, P < 0.05)*.

## Discussion

In the present study, we have characterized for the first time CBR control of HR and blood pressure in AA subjects during exercise. The primary findings are that the CBR-mediated reductions in HR and MAP in response to NS (simulated carotid hypertension) were smaller in AAs compared to age, BMI, and fitness-matched CA subjects during rest and dynamic leg exercise. These findings indicate impairment in the ability of AAs to buffer acute hypertension at rest, and during steady-state exercise.

We previously demonstrated reduced HR responsiveness to separate 5-s trials of NS among AAs at rest compared to CA subjects (Holwerda et al., 2011). Consistent with these findings, we demonstrate in the present study attenuated maximal HR responses to NS in AAs at rest (Figure 1), and extend these findings with an observed attenuation of maximal HR responses to NS during exercise (Figure 2). An attenuated minimum response (*A*_4_), saturation point, and magnitude of OP-CP detected with the carotid-cardiac function curves coupled with a trending reduction in the responding range (*A*_1_) also support the limited carotid-cardiac response to hypertensive stimuli among AA subjects compared to CA subjects (Table 2). That is, CA subjects operated further from saturation, providing a greater ability to reduce HR, despite similar maximal and operating gain. While a reduction in CBR gain can lead to inappropriate neural cardiovascular responses to exercise, racial differences in CBR-mediated reductions in HR do not appear to be due to an impaired CBR gain. Impaired CBR-mediated reductions in HR among AA subjects is more likely attributed to other neural mechanisms that influence vagal and/or sympathetic activity, as altered control of vagal activity and impaired CBR-mediated reductions in sympathetic activity are both potential contributors to the blunted HR response to NS observed in AAs.

In addition to the impaired HR responses to NS, we observed attenuated CBR-mediated maximal MAP responses to NS in AAs at rest and during exercise when compared to CAs (Figures 1, 2). Despite seemingly small differences, the attenuated MAP responses to NS among AAs may extend to a physiologically meaningful distinction. The arterial baroreflex relies significantly on changes in vasomotor activity to regulate blood pressure (Collins et al., 2001; Ogoh et al., 2003b), thus the findings in the present study suggest that AAs potentially have a diminished ability to withdraw sympathetic outflow to the vasculature during hypertensive stimuli. While BP is a function of cardiac output, the reduced bradycardic response may contribute to the attenuated MAP responses to NS seen in this group. However, although not reported, the latency for the maximum HR response to NS rarely, if at all, coincided with the latency for the maximal MAP response to NS, consistent with previous observations (Fisher et al., 2009). Thus, altered arterial baroreflex control of the vasculature in AAs likely contributes to the observed group differences in the maximal MAP response to NS.

On the other hand, no racial differences in maximal MAP responses to NP were detected at rest or during exercise. Although not measured in the current study, Ray and Monahan (2002) reported smaller changes in MSNA in response to lower-body negative pressure were associated with similar peripheral vascular responses in AA compared to CA, indicating potentially greater transduction from neural to end-organ responses. As well, other investigations have reported exaggerated pharmacologically-induced alpha-adrenergic responses in AA (Kelsey et al., 2010, 2012). A divergence between AAs and CAs in sympathetic vascular transduction is indeed an important consideration when comparing baroreflex control of blood pressure between groups. Future studies that include simultaneous assessment of vascular conductance and MSNA during CBR activation at rest and exercise are warranted.

The importance of examining the ability of the arterial baroreflex to respond to rises and falls in blood pressure separately has previously been discussed (Studinger et al., 2009; Fisher et al., 2010; Holwerda et al., 2011). In addition to CBR function curves, we provide an analysis of maximal HR and MAP responses to the separate 5-s trials of NP and NS. No racial differences in carotid-cardiac or carotid-vasomotor function curve parameters during exercise were detected. However, despite similar CBR gain, AAs demonstrated significantly smaller maximal HR and MAP responses to NS at rest and exercise, and no differences were observed in the maximal responses to NP. An examination of maximal responses to NP and NS unveiled important racial differences in acute buffering of hypertensive vs. hypotensive stimuli that may have otherwise been concealed by analysis of CBR function curves alone.

Previous studies have reported racial differences in BP responses to dynamic exercise (Alpert et al., 1981; Thomas et al., 1987; Ekelund et al., 1990; Walker et al., 1992). Statistical differences between groups in blood pressure changes from rest to exercise in the present study were not observed. However, upon removing one CA subject with an outlying BP response to exercise (MAP, Δ43 mmHg) compared to the rest of the CA group (MAP, Δ22 ± 2.7 mmHg), a greater change in MAP was observed among AAs compared to the CAs (*P* = 0.039). The tendency for an exaggerated exercise-induced change in MAP among AAs was observed despite the relatively small total subject number compared to the vast total subject numbers seen in previous reports of racial differences in BP responses to dynamic exercise (Alpert et al., 1981; Thomas et al., 1987; Ekelund et al., 1990).

The use of 5-s trials of NP and NS used in the present study potentially elicits only an abbreviated range of HR and MAP responses. Although carotid baroreceptor stimulation with 5-s trials of NP and NS in the present study was sufficient to unveil group differences, perturbation of ~20 s trials has previously been demonstrated to be required to develop a full response for MAP (Ogoh et al., 2003a). However, administration of 5-s periods of NP and NS eliminates pressure changes that would otherwise be sensed by aortic and cardiopulmonary baroreceptors, thus allowing for only carotid and, very likely, arterial baroreflex-mediated changes in HR and blood pressure to be observed.

### Perspectives

A convincing body of evidence indicates augmented blood pressure responses to emotional and physical stressors in AAs compared to CAs (Light et al., 1987; Anderson et al., 1988; Treiber et al., 1990; Calhoun et al., 1993; Terrell and Manuck, 1996; Calhoun and Mutinga, 1997; Barnes et al., 2000; Kelsey et al., 2000; Bond et al., 2001; Arthur et al., 2004), and genetic studies have attempted to described the association between such sympathetic reactivity and the development of hypertension among this population. Alpha-adrenergic receptor gene polymorphisms (Kelsey et al., 2012) and genetic variations in beta-adrenergic receptors (Kelsey et al., 2010) have recently been associated with cardiovascular reactivity among AAs. Additional mechanisms linked to risk for hypertension among AAs include elevated salt sensitivity (Sowers et al., 1988) via altered amiloride-sensitive epithelial sodium channel (ENaC) function (Ambrosius et al., 1999; Pratt et al., 2002), and small nuclear polymorphisms associated with blood pressure (Adeyemo et al., 2009; Fox et al., 2011). While the relationship between risk factors such as adrenergic receptor variations or altered ENaC function and the development of hypertension among AAs has been considered, additional studies are warranted to determine whether impaired CBR responsiveness is indeed a neural cardiovascular risk factor in this population.

In summary, our findings suggest impairment in CBR ability to defend against a hypertensive challenge among AAs during exercise compared to their CA counterparts. Despite similar gain and magnitude of resetting of the carotid-cardiac and carotid-vasomotor logistic function curves, maximal HR and MAP responses to NS (simulated hypertension) were reduced in AA subjects. The reduction in maximal HR responses to NS observed in AA subjects are likely attributed to altered control of vagal activity, while the mechanisms associated with the reductions in the peak MAP responses to NS are less discernable. Given that an intact arterial baroreflex is important for appropriate cardiovascular responses to exercise, these findings lend insight to the potential mechanism(s) responsible for previously observed racial differences in cardiovascular responses to physical and mental stimuli observed among AAs.

### Conflict of interest statement

The authors declare that the research was conducted in the absence of any commercial or financial relationships that could be construed as a potential conflict of interest.
